# EXCLUSION FROM MULTIDIMENSIONAL CIVIC ENGAGEMENT OF DIVERSE OLDER ADULTS: A PAN-EUROPEAN PERSPECTIVE

**DOI:** 10.1093/geroni/igad104.1672

**Published:** 2023-12-21

**Authors:** Fredrica Nyqvist, Sarah Dury

## Abstract

Despite decades of research on social exclusion, the understanding of the intersection between ageing and exclusion from civic engagement remains limited, both theoretically and empirically. Moreover, the research on civic engagement, including volunteering or political participation, has received unequal attention in research and the study of multidimensional civic engagement has scarcely been addressed. This symposium therefore brings together qualitative and quantitative research on multidimensional civic engagement in later life, as a part of the pan-European CIVEX-project, devoted to critically assess multidimensional civic engagement of diverse and potentially marginalized groups of older adults in a European context. The objective is to explore the main dimensions of exclusion from multidimensional civic engagement in later life and older adults’ experiences of such exclusion. Toon Vercauteren starts with a study using data from the European Quality of Life Survey (EQLS) in 33 European countries to examine multidimensional civic engagement of older adults in Europe and its key risk and support factors. Bas Dikmans will use a life course perspective to study features of exclusion from civic engagement in disadvantaged neighbourhoods in Belgium, using data collected within the international CIVEX-project. Subsequently, Fredrica Nyqvist will present results based on EQLS data on social capital and political participation in younger as well as older adults. Sandra Torres will close the symposium with a presentation on civic engagement amongst older migrants based on interview data collected in Belgium, Finland, Spain and Sweden.

